# Chronic Idiopathic Ulcers Mimicking Cecal Carcinoma: A Case Report

**DOI:** 10.7759/cureus.57792

**Published:** 2024-04-07

**Authors:** Ali Z Ansari, Sahar Hafeez, Joel Jung, Srihita Patibandla, Peter S Kim, Michael Coffin, Alex Nguyen, Kurt Kratz

**Affiliations:** 1 Department of Pathology, William Carey University College of Osteopathic Medicine, Hattiesburg, USA; 2 Department of Internal Medicine, William Carey University College of Osteopathic Medicine, Hattiesburg, USA; 3 Department of Pathology, Merit Health Wesley, Hattiesburg, USA

**Keywords:** (ibd) inflammatory bowel disease, peptic ulcer disease (pud), giant cells, lower right quadrant pain, lymphoid hyperplasia, stellate cells, stromal cells, cecal cancer, colon ulcer, chronic ulcer

## Abstract

Chronic idiopathic ulcers of the colon pose diagnostic challenges due to their elusive etiology and potential resemblance to other intestinal pathologies, such as cecal carcinoma. This case report outlines the clinical course of a 68-year-old female patient who presented to the emergency department (ED) with persistent right lower quadrant pain. Despite multiple hospital visits yielding varied diagnoses, a definitive diagnosis was only made following a laparoscopic partial colectomy, which revealed chronic idiopathic ulcers with transmural scarring and adhesions to adjacent small intestine loops. Histological examination demonstrated a substantial ulcer bed populated by inflammatory cells, including large stellate and spindled stromal cells within the granulation tissue, alongside lymphoid hyperplasia and scar tissue extending into the muscularis propria. The initial presentation of this case could easily be mistaken for appendicitis, diverticulitis, carcinoma, or irritable bowel syndrome, highlighting the significance of considering chronic idiopathic ulcers in the differential diagnosis of patients presenting with cecal masses.

## Introduction

Chronic idiopathic ulcers affecting the small bowel and colon represent rare and challenging clinical scenarios, characterized by persistent mucosal ulceration without a discernible cause [1]. Diagnosis and management are often intricate due to the diverse array of presenting symptoms. While the precise etiology and pathogenesis are poorly understood, existing literature suggests potential involvement of immune-mediated responses, infectious agents, non-steroidal anti-inflammatory drug (NSAID) use, or a combination thereof [2]. These ulcers may also be associated with various underlying conditions, including Crohn's disease, celiac disease, tuberculosis, typhoid fever, and immunodeficiency disorders [3]. Additionally, the range of potential causes considered in the differential diagnosis includes infections like tuberculosis and *Yersinia enterocolitica* [4]. Histopathologically, chronic idiopathic ulcers manifest as persistent and extensive mucosal ulceration accompanied by granulomatous inflammation, further complicating the diagnostic process. Typically, diagnosis relies on exclusion criteria, ruling out common factors such as *Helicobacter pylori* infection, NSAID use, and inflammatory bowel disease (IBD). Patients often present with abdominal pain, nausea, and vomiting, necessitating accurate differentiation from other conditions to avert potential complications such as perforation, obstruction, and fistula formation, which can lead to significant morbidity and mortality [5].

Recent studies have highlighted a global increase in chronic idiopathic ulcers, paralleled by an increasing incidence of peptic ulcer disease (PUD) unrelated to *H. pylori* infection or NSAID use [6]. Due to the rarity of the condition and the absence of standardized treatment protocols, the definitive management and prognosis remain uncertain. Chronic idiopathic ulcers of the colon often rank lower on the list of differentials, as they are typically surpassed by more prevalent conditions like PUD, colonic carcinoma, Crohn's disease, and other gastrointestinal disorders. A study has indicated that chronic idiopathic ulcers exhibit a higher recurrence rate compared to *H. pylori*-positive and NSAID-induced PUD over a five-year period. Notably, idiopathic ulcers have been identified as an independent risk factor for ulcer recurrence [7]. This case report emphasizes the critical need for thorough evaluation in patients exhibiting gastrointestinal symptoms, particularly when chronic idiopathic ulcers are among the potential differentials.

## Case presentation

A 68-year-old non-smoking female with a medical history notable for coronary artery disease (CAD), diabetes mellitus, diverticulitis, and arthritis presented to the emergency department (ED) reporting severe abdominal pain localized to the right lower quadrant. She states that this has been ongoing for approximately 16 hours with multiple episodes of nausea and vomiting. Initial assessment prompted a computed tomography (CT) scan of the abdomen and pelvis with contrast, along with a complete blood count (CBC) and comprehensive metabolic panel (CMP). Laboratory results revealed a hemoglobin level of 10.5 g/dL, white blood cell count of 9,500 cells/µL, platelet count of 280,000/µL, blood glucose of 110 mg/dL, electrolytes within normal limits, normal renal function, and liver enzymes within normal range. The patient denied constitutional symptoms, changes in bowel habits, hematochezia, or hematemesis. While there was a familial history of heart disease, there were no reported gastrointestinal disorders within the family.

**Figure 1:**
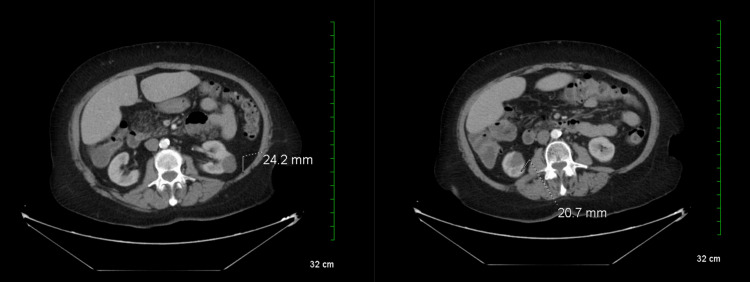
CT scan (abdomen) CT scan images reveal bilateral renal cysts. The cyst on the left measures 2.4 cm, while the cyst on the right measures 2.1 cm. CT: Computed tomography

The CT scan revealed multiple colonic diverticula in the sigmoid colon without signs of inflammation, bilateral simple renal cysts, an old T12 compression fracture, an absent gallbladder, and an atrophic pancreas (Figure 1). Additionally, a grade 1 anterolisthesis of L4 on L5 was noted. Following discharge, the patient was prescribed Zofran® (ondansetron) 4 milligrams and advised to follow up with her primary care physician (PCP).

During a subsequent visit, the PCP raised concerns regarding the possibility of colon cancer, due to unintentional weight loss, prompting a referral to a general surgeon for further evaluation. The diagnostic process included a colonoscopy, which identified a mass within the cecum. The general surgeon suspected primary colon carcinoma and recommended surgical intervention, specifically a resection. Additionally, a laparoscopic partial colectomy with end colostomy and closure of the distal segment was advised. Shortly thereafter, the patient underwent a laparoscopic right colectomy. The surgical specimen obtained measured 11 x 3.5 cm and included the appendix, which measured 5.5 x 1.0 cm, along with a segment of adhered ileum. Upon gross examination, an ulcerated tumor mass measuring 5.5 x 9.0 x 2.0 cm was observed near the appendiceal orifice within the cecal specimen. No mucosal abnormalities were identified in the adhered segment of the small bowel.

**Figure 2:**
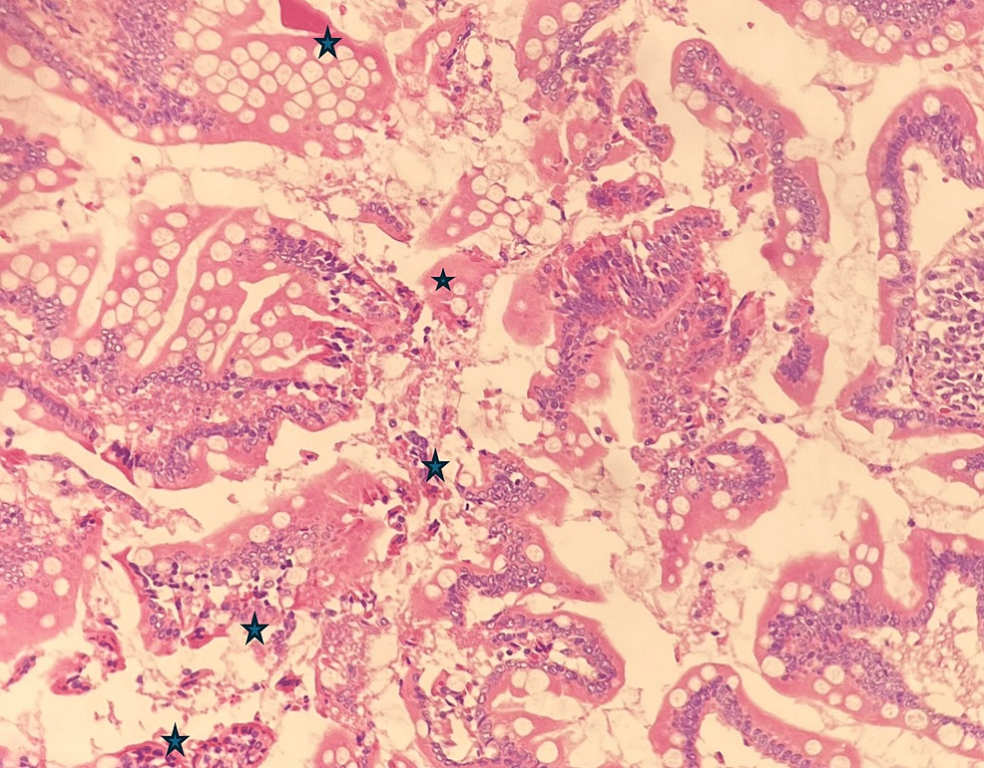
Histopathology photomicrograph I (200X magnification) The image is depicting ulceration in a cecal mass, characterized by severe epithelial destruction, crypt distortion, and notable deposits of fibrin and hemosiderin within the lamina propria (black stars).

The microscopic examination of the tumor mass revealed extensive mucosal ulceration characterized by patchy overlying purulent exudate and hemosiderin deposits (Figure 2).

**Figure 3:**
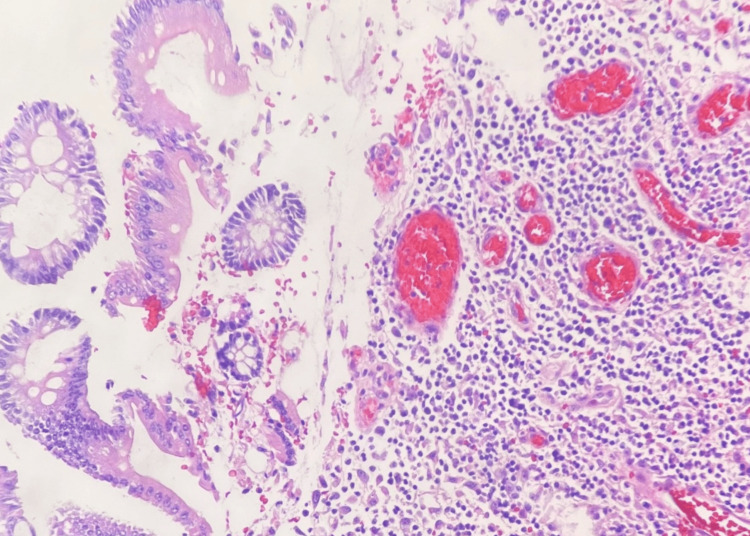
Histopathology photomicrograph II (200X magnification) The photomicrograph is revealing granulomatous tissue in the ulcer bed, characterized by abundant lymphocytes, scattered eosinophils, and prominent neutrophils.

**Figure 4:**
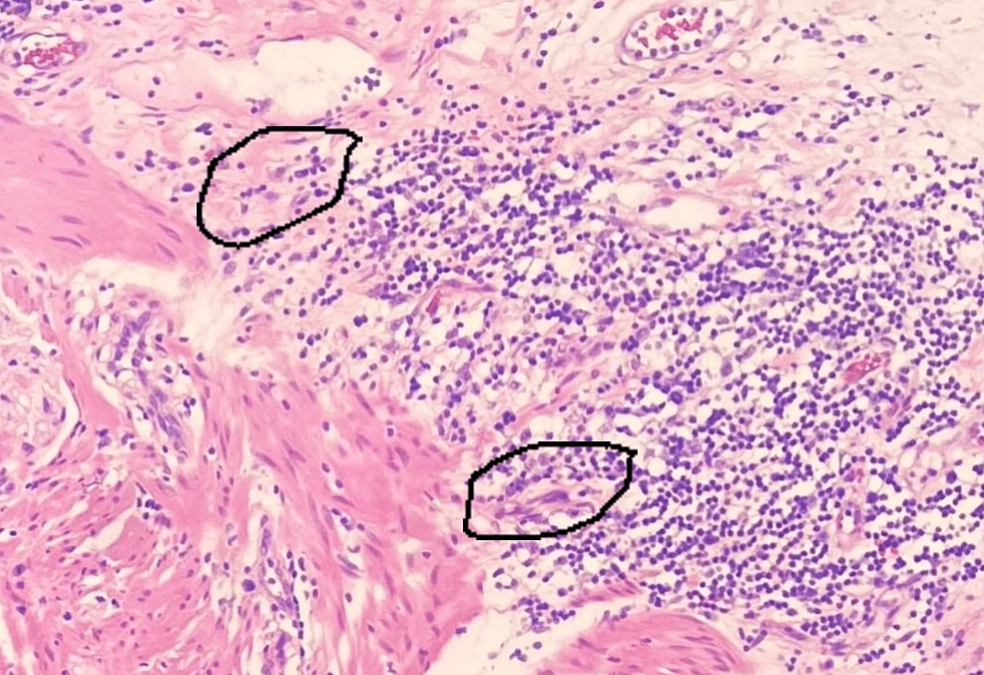
Histopathology photomicrograph III (200X magnification) The photomicrograph is displaying granulomatous tissue within the ulcer bed, highlighting the presence of stellate and spindled cells (encircled), potentially indicating muscle and neurological dysfunction.

Within the ulcer bed, pronounced lymphoplasmacytic and granulomatous inflammation was evident, marked by a substantial presence of lymphocytes alongside an admixture of neutrophils, eosinophils, and prominent stellate stromal cells (Figures 3, 4).

**Figure 5:**
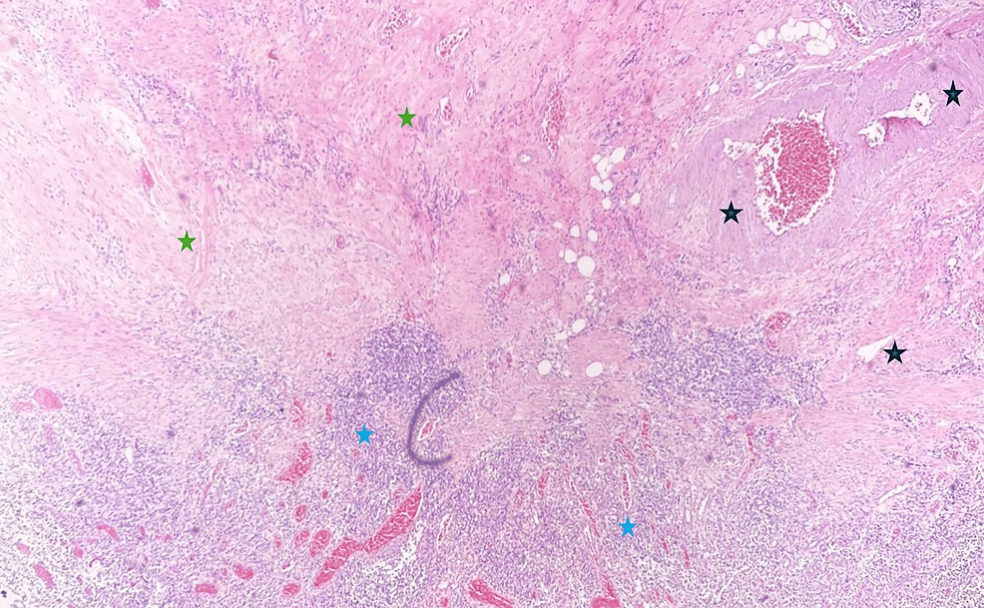
Histopathology photomicrograph (20X magnification) The image is illustrating the resected colon wall, revealing submucosal fibrosis (green stars), marked lymphocytic infiltration (blue stars), and abundant thick-walled vessels (black stars).

**Figure 6:**
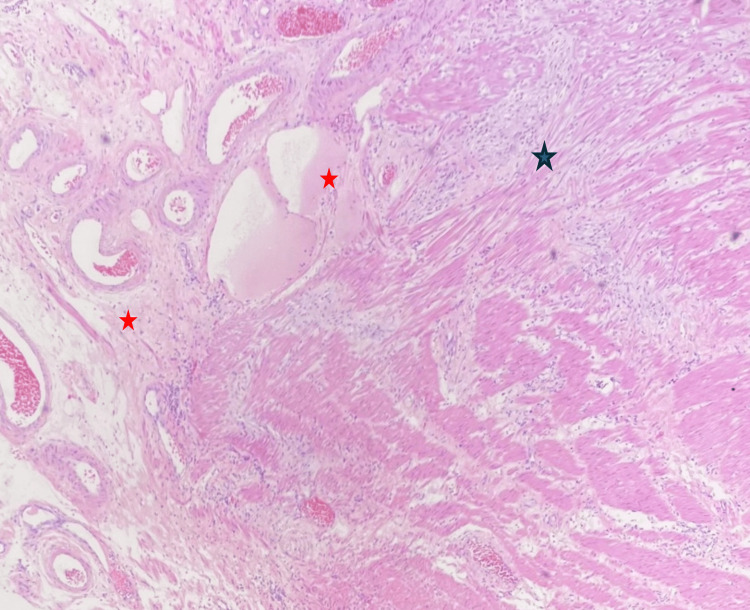
Histopathology photomicrograph of the resected colon wall The image is revealing extensive scar tissue formation (black star) within the muscularis propria, along with a notable presence of serous exudate (red stars) leading to adhesion formation.

**Figure 7:**
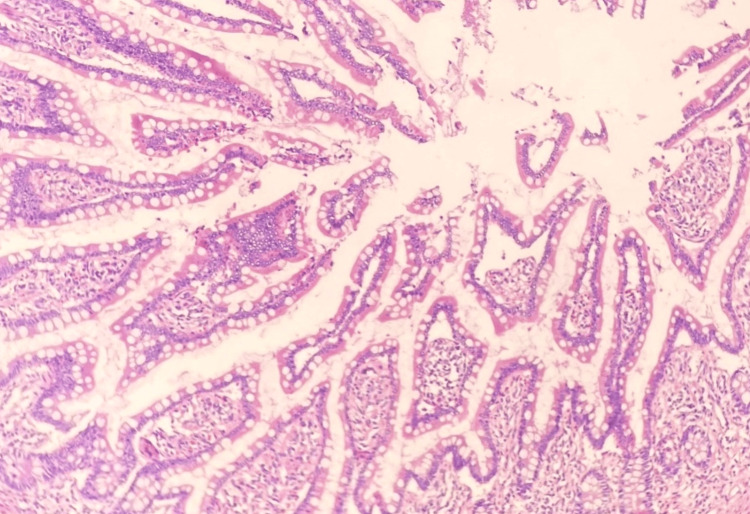
Histopathology photomicrograph III (200X magnification) This photomicrograph at 200x magnification reveals an unaffected segment of a resected colon specimen. The preserved mucosal integrity, uniform nuclear alignment in the columnar epithelium, and intact basement membrane are shown.

In the underlying submucosa, signs of chronic inflammation were apparent, including marked lymphocytic infiltration, fibrosis, and numerous thick-walled vessels (Figure 5). Notably, submucosal fibrosis extended through the overlying muscularis propria, resulting in extensive scar tissue and serous exudation within the muscular layer and serosa (Figure 6). Although the mucosa of the appendiceal orifice exhibited signs of inflammatory changes, the appendix itself appeared normal. No dysplastic changes were noted in the mucosa of other segments of the colocecal specimen (Figure 7). Peri-colonic lymph nodes displayed no remarkable features. Special stains for acid-fast, fungal, or bacterial organisms, including periodic acid-Schiff (PAS), Gomori methenamine silver (GMS), and Gram stains, yielded negative results for pathogenic organisms. In summary, these findings indicate a case of chronic idiopathic isolated colonic ulcer with transmural extension of scar tissue, resulting in adhesion formation with the adjacent loop of small bowel. Crohn's disease was considered, however, the absence of symptomatic manifestations in the patient did not support a likely diagnosis.

## Discussion

Idiopathic colonic ulcers, commonly referred to as nonspecific idiopathic colonic ulcers (NSICU), isolated ulcers of the large intestine, or solitary ulcers, represent a distinct clinical presentation characterized by the absence of associated underlying colitis. They are rarely encountered as incidental findings during routine screening colonoscopies [8]. In this case, the patient's age, gender, and initial colonoscopic findings are consistent with the typical characteristics associated with NSICU. These ulcers can manifest in individuals across all age demographics, with a higher incidence observed among those in their fifth and sixth decades, displaying a slight female predilection [9]. Over 50% of NSICU cases exhibit involvement on the right side of the colon, demonstrating a particular inclination toward the cecum in close proximity to the ileocecal valve [10]. As seen in this case, macroscopic examination of the cecocolic specimen revealed an isolated, well-defined ulcerated mass without abnormal thickening in the surrounding colonic or appendiceal mucosa, an observation classically linked with Crohn's disease. This finding corresponds with prior literature indicating that NSICU typically manifests as a single lesion on imaging, characterized by rounded contours with sharply delineated borders along the antimesenteric wall, bordered by intact colonic mucosa [11].

Histopathological analysis of this case revealed acute inflammation, mucosal sloughing, and extensive fibrosis spanning the submucosa and muscularis propria of the cecal wall, confirming the chronic nature of the ulcer. Although granulation tissue beneath the ulcer bed exhibited epithelioid cells, the absence of giant cells and Paneth cell hyperplasia did not support a diagnosis of IBD-associated ulceration [12]. Notably, solitary idiopathic colonic ulcers have been associated with obstruction due to extensive scar tissue formation. However, in this instance, despite transmural scarring, the patient did not experience colonic obstruction, likely due to the fibrosis involving the antimesenteric wall of the cecum, positioned outside the luminal flow. Given that a subset of chronic idiopathic ulcers may arise from ischemic injury or drug-induced causes, a thorough examination is essential to rule out various differentials, as the diagnosis significantly influences treatment strategies. In this case, despite comprehensive evaluation, the ulcer remained idiopathic, with no identifiable causative or risk factors established. Considering the patient's age of approximately 70 years and a history of CAD, speculation arises regarding ischemic injury secondary to arteriosclerosis of the cecal artery as a potential contributor to ulcer formation. Another plausible factor is the chronic use of NSAIDs, supported by lower lumbar anterolisthesis evident in the patient's CT scan [13].

The patient's favorable outcome is highlighted by her presentation to the ED, potentially in the acute phase of the ulcer, which mimicked signs of appendicitis. Given that cecal/colonic perforation represents the most prevalent complication of NSICU during its acute phase, timely and appropriate management of this case averted such an eventuality. It is important to note that the clinical presentation of NSICU can mimic various conditions, such as appendicitis or carcinoma, posing challenges for preoperative diagnosis. However, suspicion of a nonspecific ulcer arises when endoscopic biopsy results fail to identify other causative factors.

## Conclusions

This case highlights the diagnostic complexity associated with chronic idiopathic ulcers, particularly when presenting in the context of acute abdominal pain. Despite multiple hospital visits and diagnostic evaluations, a definitive diagnosis in this case was only achieved following laparoscopic partial colectomy. The histopathological examination revealed characteristic features of chronic idiopathic ulcers, emphasizing the necessity of considering this condition in the differential diagnosis of patients presenting with cecal masses. Furthermore, this case highlights the importance of thorough evaluation and vigilant consideration of less common etiologies, as timely recognition and appropriate management are crucial in averting potential complications associated with chronic idiopathic ulcers, including perforation and obstruction. Further research is warranted to elucidate the underlying mechanisms and optimal management strategies for this challenging clinical condition.
